# A Method for 6D Pose Estimation of Free-Form Rigid Objects Using Point Pair Features on Range Data

**DOI:** 10.3390/s18082678

**Published:** 2018-08-15

**Authors:** Joel Vidal, Chyi-Yeu Lin, Xavier Lladó, Robert Martí

**Affiliations:** 1 Department of Mechanical Engineering, National Taiwan University of Science and Technology, Taipei 106, Taiwan; jerrylin@mail.ntust.edu.tw; 2 Computer Vision and Robotics Institute, University of Girona, 17003 Girona, Spain; xavier.llado@udg.edu (X.L.); robert.marti@udg.edu (R.M.); 3 Taiwan Building Technology Center, National Taiwan University of Science and Technology, Taipei 106, Taiwan; 4 Center for Cyber-Physical System Innovation, National Taiwan University of Science and Technology, Taipei 106, Taiwan

**Keywords:** computer vision, range data, 6D pose estimation, 3D object recognition, scene understanding, model-based vision

## Abstract

Pose estimation of free-form objects is a crucial task towards flexible and reliable highly complex autonomous systems. Recently, methods based on range and RGB-D data have shown promising results with relatively high recognition rates and fast running times. On this line, this paper presents a feature-based method for 6D pose estimation of rigid objects based on the Point Pair Features voting approach. The presented solution combines a novel preprocessing step, which takes into consideration the discriminative value of surface information, with an improved matching method for Point Pair Features. In addition, an improved clustering step and a novel view-dependent re-scoring process are proposed alongside two scene consistency verification steps. The proposed method performance is evaluated against 15 state-of-the-art solutions on a set of extensive and variate publicly available datasets with real-world scenarios under clutter and occlusion. The presented results show that the proposed method outperforms all tested state-of-the-art methods for all datasets with an overall 6.6% relative improvement compared to the second best method.

## 1. Introduction

Pose estimation is a major part for an efficient and flexible object inspection, grasping or manipulation system. Since the origins of visual image processing, object detection and localization have been considered as an essential part of the object recognition and scene understanding problems, representing one of the main motivations and research directions in the computer vision field [1]. Historically, 6D pose estimation of free-form objects, also referred to as sculptured objects, has been a challenging research goal due to the variability of the vision systems and the three-dimensional nature of the problem [2]. The introduction of range data applied to object recognition in the late 1970s [3] opened a new research path by providing additional depth information and data sources robust to illumination changes. Before the 1990s, most of the approaches were focused on the detection of objects within a specific narrow domain of simple solids, polygonal or polynomial shapes [4]. Since then, the increasing computational power and the introduction of more accessible sensor technologies [5] have motivated new branches and research directions, continuously enlarging the object domain and the complexity of the scenes. Nowadays, the most prominent research directions can be categorized in feature-based, template matching and machine learning methods.

Feature-based methods can be considered the most extended solution to approach the object recognition problem using three-dimensional data. They are commonly divided in two groups: local and global methods. Local feature-based methods [6] are based on matching descriptors of local surface characteristics, usually extracted around selected keypoints for efficiency reasons. Among their principal attributes, there is the implicit robustness against occlusion and clutter resulting from the local nature of the description. On the other hand, global feature-based methods (e.g., [7,8,9,10]) follow a different pipeline for which the whole object surface is described by a single or small set of descriptors. For most approaches, each global feature describes each of the views of the object, named view-dependent descriptors. The global nature of these descriptors implies the separation of the described surface from their surroundings, which introduces a segmentation step on the common global feature recognition pipeline.

In another direction, template matching techniques, extended from two-dimensional computer vision, have also been proposed for RGB-D data. For two-dimensional images, approaches relaying on image gradients [11,12] have provided relatively good results under occlusion and illumination changes. Based on these methods, Hinterstoisser et al. [13] proposed a template matching technique extended to RGB-D data using quantized surface normals as a depth cue. In a similar fashion, recently, Hodan et al. [14] applied the concept of multimodal matching of [13] on an efficient cascade-style evaluation strategy.

Techniques based on supervised machine learning [15,16] have been also used for object recognition and pose estimation on RGB-D data. Brachmann et al. [17] introduced a method and an extension [18] for object pose estimation using a random forest to classify the pixels of an RGB-D image. Tejani et al. [19] adapted the multimodal template of [13] as a scale-invariant patch representation integrated into a random forest. Finally, Kehl et al. [20] presented a method based on Convolutional Neural Network (CNN) using RGB-D patches.

In 2010, a feature-based method based on Point Pair Features [21] was presented as a compromised solution between the local and global approaches. Following the traditional local matching pipeline while using a global modeling, the approach showed a promising trade-off between recognition rates and speed. Based on the idea of surflet pairs [22], the method relays on four-dimensional features defined between pairs of oriented points to describe the object surface. These features are used to find correspondences between scene and model pairs of points, which are grouped for each scene point, generating a set of pose candidates. The correspondences are determined by quantizing the feature space, effectively grouping similar pairs together. Then, for each scene point, a candidate pose is generated by grouping all related corresponding pairs on a two-dimensional space defined by the model corresponding points and the rotation angles around the points normal. At the end, candidate poses are clustered and sorted to obtain a final hypothesis.

Following this idea, in 2011, Kim and Medioni [23] proposed a variation of the original method by including visible context information, differentiating visible points, points on the surface and invisible points. In addition, they used two novel verification steps to check for surface alignment and surface separability. Drost and Ilic [24] introduced in 2012 a multimodal extension of the method including edge information extracted from RGB data. Moreover, their approach included non-maximum suppression of the clustered poses and a pose refinement step based on Iterative Closest Point (ICP). Birdal and Ilic [25], in 2015, analyzed some drawbacks of the method and proposed a novel framework to overcome some of the issues regarding the high dimensionality of the search space, sensitivity of the correspondence and the effect of outliers and low density surfaces. Their novel solution included a coarse-to-fine segmentation step, a weighted Hough voting and a fast ranking and verification postprocessing steps. Hinterstoisser et al. [26] published in 2016 a major revision of the method presenting a novel approach with higher performance and improved robustness against occlusion and clutter. Among their contributions, they proposed to use normal information during preprocessing and mitigated the discretization problems of the data by an exhaustive neighbor checking. Their method used two different size voting zones and an additional data structure to avoid multiple voting of similar pairs. In addition, they proposed an improved bottom-up clustering strategy and several additional verification steps. Recently, Kiforenko et al. [27] presented a complete performance evaluation of the Point Pair Features including a detailed comparison with the most popular local features.

This paper extends the preliminary work presented in [28], defining a novel method based on the Point Pair Features voting approach [21] for robust 6D pose estimation of free-form objects under clutter and occlusions on range data. The contributions of the proposed method are novel preprocessing steps to extract relevant point cloud data, a more efficient feature matching approach, which mitigates quantization errors, and an improved hierarchical clustering step. In addition, this is complemented with several postprocessing steps, including a novel view-dependent re-scoring process for candidate hypotheses and efficient verifications steps to discard false-positive cases. These processes are presented in an integrated local feature-based pipeline divided in six consecutive steps. Finally, the proposed method is tested against other 15 state-of-the-art methods on the recently presented BOP benchmark [29] obtaining a relative improvement of 6.6% with respect to the second best method.

## 2. Method

### 2.1. The Basics

First introduced by Drost et al. [21], the Point Pair Features voting approach is a feature-based solution combining a global modeling and a local matching stage within a local pipeline using sparse features. The method details have been explained in several publications (e.g., [21,26]), however, for completeness and better understanding of the paper, we consider it important to offer a general overview of the approach with special emphasis on some specific points.

**Figure 1:**
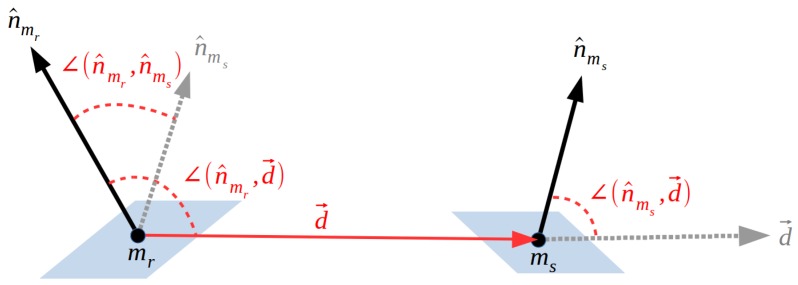
The Point Pair Feature definition for a model’s point pair $\left(m_{r},m_{s}\right)$.

Using point cloud representations of oriented points (i.e., points with normals), the method relays on four-dimensional features extracted from pairs of points (from now on “point pairs” or simply “pairs”) to globally describe the whole object from each surface point in a way that later the object can be locally matched with the scene. This four-dimensional feature, called Point Pair Feature or PPF, defines an asymmetric description between two oriented points by encoding their relative distance and normal information, as shown in Figure 1. In detail, having a set of points in the 3D space $M\subset R^{3}$ representing the model object, for a given 3D point $m_{r}\in M$, called reference, and a given 3D point $m_{s}\in M$, named second, such that $m_{r}\neq m_{s}$, with their respective unit normal vectors ${\hat{n}}_{m_{r}}$ and ${\hat{n}}_{m_{s}}$, a model four-dimensional feature $f^{m}\in \left(F^{m}\subset R^{4}\right)$ is defined by Equation (1),

(1) $$F_{rs}\left(m_{r},m_{s},{\hat{n}}_{m_{r}},{\hat{n}}_{m_{s}}\right)=\left[\;|\right|\vec{d}||,∠\left({\hat{n}}_{m_{r}},\vec{d}\right),∠\left({\hat{n}}_{m_{s}},\vec{d}\right),∠\left({\hat{n}}_{m_{r}},{\hat{n}}_{m_{s}}\right){\;]}^{T},$$

where $\vec{d}=\left(m_{s_{x}}-m_{r_{x}},m_{s_{y}}-m_{r_{y}},m_{s_{z}}-m_{r_{z}}\right)$ and $∠(\vec{a},\vec{b})$ is the angle between the vector $\vec{a}$ and $\vec{b}$. In the same way, having a set of point $S\subset R^{3}$ representing the scene data, the function $F_{rs}$ can be applied to compute a scene PPF using a pair of scene points $s_{r},s_{s}\in S$ such that $s_{r}\neq s_{s}$, with their respective unit normal vectors ${\hat{n}}_{s_{r}}$ and ${\hat{n}}_{s_{s}}$. Notice that, if the object model has |M| points, the total number of features is defined by $|F^{m}{\left|=|M\right|}^{2}-\left|M\right|$. In order to reduce the effect of this square relation on the method performance, the input data of both model and scene are downsampled with respect to the model size, effectively decreasing the complexity of the system.

The method can be divided into two main stages: modeling and matching. On modeling, the global model descriptor is created by computing and saving all the possible model pairs with their related PPF. During the matching stage, the model pose in the scene is estimated by matching the scene pairs with the stored model pairs using the PPF. This matching process consists of two distinctive parts: (1) find the correspondence between the pairs using the four-dimensional features and (2) group the correspondences generating hypotheses’ poses.

**Figure 2:**
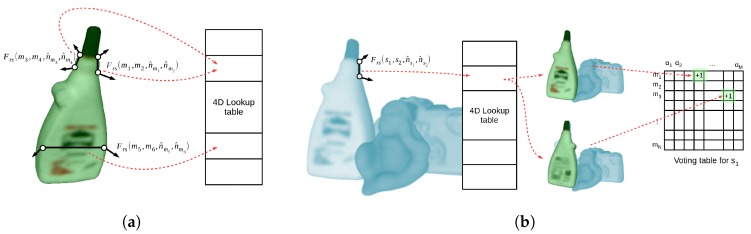
Representation of the modeling and matching steps of the Point Pair Features voting method. (**a**) modeling example for three point pairs from the model; (**b**) matching example for one point pair from the scene.

The correspondence problem between similar point pairs is efficiently solved by grouping the pairs with the same quantized PPF on a hash table or, alternatively, a four-dimensional lookup table. Quantizing the feature space defines a mapping from each four-dimensional space element to the set of all point pairs that generate this specific feature. In particular, for the object model, this mapping from quantized features to sets of model pairs defines the object model description expressed by the function $L:Z^{4}\to P\left(M_{\mathrm{pp}}\right)$, where $M_{\mathrm{pp}}=\left\{\left(m_{r},m_{s}\right)|m_{r},m_{s}\in M,m_{r}\neq m_{s}\right\}$ and P(X) represents the power set of X. In other words, point pairs that generate the same quantized PPF are grouped together on the same table position pointed by their common quantized index, effectively grouping pairs with similar features. This process of model construction is done during the modeling stage, as shown in Figure 2a for three sample point pairs. Using this model description, given one scene pair, similar model pairs can be retrieved by accessing a table position pointed by the PPF quantized index. The quantization index is obtained by a quantization function $Q:R^{4}\to Z^{4}$ using the step size $\Delta_{dist}$ for the first dimension and $\Delta_{angle}$ for the remaining three dimensions. The quantization step size will bound the similarity level, i.e., correspondence distance, between matching features, and hence point pairs. Defining a function $N:R^{3}\to R^{3}$ that computes a normal from a point, the correspondence matching subset of model pairs $A\subseteq M_{\mathrm{pp}}$ for a given scene pair $\left(s_{r},s_{s}\right)$ and its related quantized feature ${\bar{f}}^{s}=Q\left(F_{rs}\left(s_{r},s_{s},\hat{n_{s_{r}}},\hat{n_{s_{s}}}\right)\right)$ is defined by Equation (2):

(2) $$L\left({\bar{f}}^{s}\right)=\left\{\left(m_{r},m_{s}\right)\in M_{\mathrm{pp}}|Q\left(F_{rs}\left(m_{r},m_{s},N\left(m_{r}\right),N\left(m_{s}\right)\right)\right)={\bar{f}}^{s}\right\}.$$

**Figure 3:**
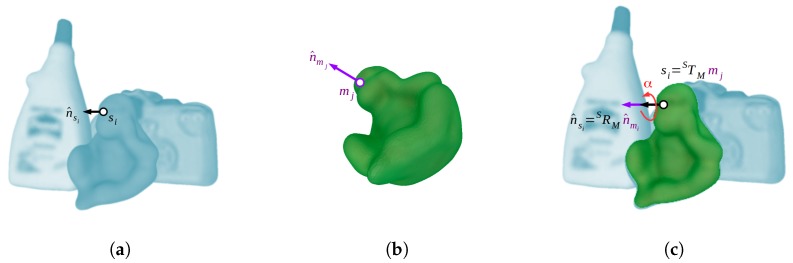
Representation of the local coordinate (LC) system used by the point pair features method; (**a**) scene oriented point; (**b**) corresponding object model oriented point; (**c**) alignment of the model with the scene by using the two oriented points and the α angle.

From each scene-model point pair correspondence, a 6D pose transformation, or hypothesis, can be generated. Specifically, for a corresponding point pair $(m_{r},m_{s})\in A$, the matched reference points $\left(s_{r},m_{r}\right)$ and their normals $\left({\hat{n}}_{s_{r}},{\hat{n}}_{m_{r}}\right)$ constrain five degrees of freedom, aligning both oriented points, and the second points $\left(s_{s},m_{s}\right)$, as long as they are non-collinear, constrain the remaining degree of freedom, which is a rotation around the aligned normals. However, the discriminative capability of a single four-dimensional feature from two sparse oriented points is clearly not enough to uniquely encode any surface characteristic, producing wrong correspondences. Therefore, the method requires a group of consistent correspondences to support the same hypothesis. Actually, the more correspondences support a single pose, the more likely this will be. In this regard, grouping consistent point pair correspondences, or, alternatively, 6D poses obtained from corresponding pairs have a high dimension complexity. In order to effectively solve this problem, a local coordinate, which we will refer to as LC, is used to efficiently group the poses within a two-dimensional space. As with two corresponding pairs, for a given scene point $s_{i}\in S$ that belongs to the object model, a 6D pose can be defined by only using one corresponding model point $m_{j}\in M$ and a rotation angle α around their two aligned normals, i.e., ${\hat{n}}_{s_{i}}$ and ${\hat{n}}_{m_{j}}$. In this way, for the scene point $s_{i}$, a 6D pose transformation candidate ${}^{S}T_{M}\in SE\left(3\right)$ can defined by the LC represented by the parameters $\left(m_{j},\alpha \right)$, as shown in Figure 3. To solve this transformation, both points and normals are aligned respectively with the origin and *x*-axis of a common world coordinate system {W}. Taking the scene point, this alignment can be expressed by the transformation ${}^{W}T_{S}=\left(R,t\right)\in SE\left(3\right)$. The rotation that aligns the normal vector ${\hat{n}}_{s_{i}}$ to the *x*-axis ${\hat{e}}_{x}$ is defined by the axis-angle representation $\theta \hat{v}$, where $\theta =∠({\hat{n}}_{s_{i}},{\hat{e}}_{x})$ and $\hat{v}=\frac{{\hat{n}}_{s_{i}}\times {\hat{e}}_{x}}{\left|\right|{\hat{n}}_{s_{i}}\times {\hat{e}}_{x}\left|\right|}$. Therefore, the rotation matrix R∈SO(3) can be efficiently found using the Rodrigues’ rotation formula [30]. In turn, the translation $t\in R^{3}$ is defined by $t=-Rs_{i}$. Exactly in the same way, the transformation ${}^{W}T_{M}\in SE\left(3\right)$ is found for the model point $m_{j}$ and its normal ${\hat{n}}_{m_{j}}$. Using these two transformations and the rotation angle, the 6D pose for a given object instance is defined by Equation (3):

(3) $${}^{S}T_{M}={{(}^{W}T_{S})}^{-1}R_{x}{\left(\alpha \right)}^{W}T_{M},$$

where $R_{x}\left(\beta \right)\in SO\left(3\right)$ represents a rotation of β angle around the *x*-axis. Using the LC, the correspondence grouping problem can be individually tackled for any scene pair created from $s_{i}$ by grouping the corresponding model pairs in a two-dimensional space using the parameters ($m_{j},\alpha$).

During grouping and hypothesis generation, for every reference scene point $s_{i}$, the method intends to find the LC, i.e., ($m_{j},\alpha$), which defines the best fitting model pose on the scene data or, in other words, that maximizes the number of pairs correspondences that support it. This correspondence grouping problem is solved by defining a two-dimensional voting table or *accumulator*, in a Generalized Hough Transform manner, representing the parameter space of the LC, where one dimension represents the corresponding model point $m_{j}$ and the other the quantized rotation angle α. In particular, for each possible scene pair generated from $s_{i}$, i.e., $\left(s_{r},s_{s}\right)\in \left\{\left(s_{k},s_{l}\right)|s_{k},s_{l}\in S,s_{k}\neq s_{l},s_{k}=s_{i}\right\}$, a LC will be defined by a corresponding pair $\left(m_{r},m_{s}\right)$ reference point, i.e., $m_{j}=m_{r}$, and the rotation angle α defined by the two second points $\left(s_{s},m_{s}\right)$. The corresponding model pairs are retrieved from the lookup table using the quantized PPF and, for each obtained LC, a vote is cast on the table, as represented by Figure 2b for a single pair. After all pairs are checked, the peak of the table represents the most supported LC, and hence the most likely pose, for this specific $s_{i}$ point. This process is applied to all or, alternatively, a fraction of the scene points, obtaining a set of plausible hypotheses.

**Figure 4:**
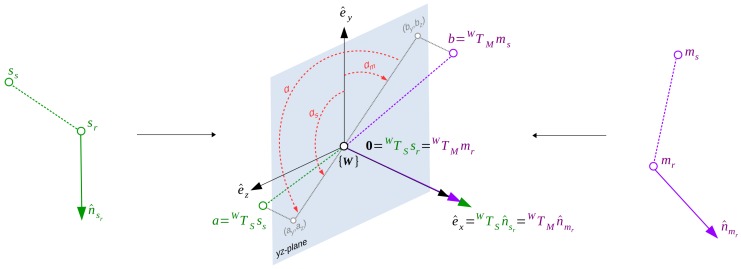
Representation of the LC α angle definition from two corresponding pairs $\left(s_{r},s_{s}\right)$ and $\left(m_{r},m_{s}\right)$.

To increase the efficiency of the voting part, which requires to compute the α angle for each pair correspondence, it is possible to split the rotation angle α in two parts; one part related to the model point, $\alpha_{m}$, and one part related to the scene point, $\alpha_{s}$. In detail, taking into account that in the intermediate world coordinate system the α angle is defined around the *x*-axis, the rotation on the two-dimensional yz-plane can be divided with respect to the positive *y*-axis. In this case, the $\alpha_{m}$ and $\alpha_{s}$ will be defined as the rotation angles between the positive *y*-axis vector ${\hat{e}}_{y}$ and the yz-plane projection of the vectors obtained by the world transformed second points of the model pair (${}^{W}T_{M}\;m_{s}$) and scene pairs (${}^{W}T_{S}\;s_{s}$). As shown in Figure 4, these angles can be defined as $\alpha_{s}=a$tan$2(a_{z},a_{y})$ and $\alpha_{m}=a$tan$2(b_{z},b_{y}),$ where $a{=}^{W}T_{S}\;s_{s}$, $b{=}^{W}T_{M}\;m_{s}$ and *a*tan2(β,γ) represents the multi-valued inverse tangent. With this solution, the model angle can be precomputed during the modeling stage and saved alongside the reference point in the lookup table $\left(m_{r},\alpha_{m}\right)$. Later, during the matching stage, for each scene pair, the α angle is computed by adding the two angles. Considering that α is defined from the model to the scene, the total angle can be computed as $\alpha =\alpha_{s}-\alpha_{m}$.

Finally, in order to join similar candidate poses generated from different scene reference points, the method is completed with a clustering approach that groups similar poses that do not vary in rotation and translation more than a threshold.

### 2.2. Our Proposal

**Figure 5:**
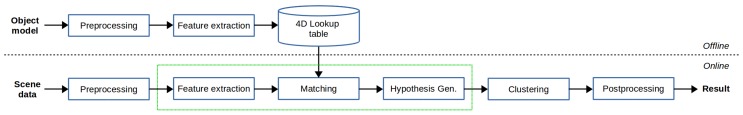
Proposed method pipeline. The green fine dotted box represents the iterative implementation of the steps *Feature Extraction*, *Matching* and *Hypothesis generation* for each scene point.

In this section, we define a new method based on the well-known Point Pair Features voting approach [21] for robust 6D pose estimation of free-form objects under clutter and occlusions on range data. In detail, the original ideas presented in [21] are improved and a complete method within a local feature-based pipeline is defined. The proposed method pipeline, shown in Figure 5, can be divided in an *Offline* modeling and an *Online* matching stages with six basic steps: *Preprocessing*, *Feature Extraction*, *Matching*, *Hypothesis Generation*, *Clustering* and *Postprocessing*. Due to this method’s particular correspondence grouping step, using a voting table for each scene point, a basic straightforward implementation will require to create a voting table for each of the scene points during the hypothesis generation step, with large memory requirements. From a practical point of view, a more efficient solution is to iteratively generate a hypothesis for each scene point using a single voting table. In this regard, the green fine dotted box in Figure 5 represents the iterative implementation of the steps *Feature Extraction*, *Matching* and *Hypothesis generation* for each scene point. The method is considered to work with mesh data for modeling and organized point cloud for matching, as standardized data types.

#### 2.2.1. Preprocessing

The Point Pair Feature voting method strongly relies on the discriminative effect of the PPF and their sparse nature to allow an efficient, structural aware local matching. The performance of the original four-dimensional PPF and its variants has been deeply studied by Kiforenko et al. [27]. Their work concludes that a set of PPF globally defining a model point has stronger discriminative capability than most local features. On the other hand, they also showed that, despite its robustness, the PPF are significantly affected by noise. In fact, individually, each feature relies on the quality and relevance of the normal and distance information extracted from the sparse surface characteristics provided by the pairs of the sampled data. In this sense, low quality or non-discriminative features will reduce speed and decrease the recognition performance of the method. Therefore, the overall global description performance, in terms of time and recognition, depends on the number of features and the relevance and quality of each individual feature. This relation makes the performance of the approach rely significantly on the preprocessing steps. In turn, the sampling and normal estimation in preprocessing are mainly affected by the sensor noise and the relative size of the underlying surface characteristics. Taking these considerations into account, we propose a combination of two normal estimation approaches and a novel downsampling methodology that mitigates sensor noise, accounts for surface variability and maximizes the discriminative effect of the features.

##### Normal Estimation

For the normal estimation problem, we propose using two different variants regarding the input data representation of each stage. For the *Offline* stage, using reconstructed or CAD mesh data, the normals are estimated by averaging the normal planes of each vertex’s surrounding triangles. In this case, noise and resolution limitations regarding surface reconstruction techniques are considered out of the scope of this manuscript, and thus not considered. For the *online* stage, using the organized point cloud data, we use the method proposed in [13], based on the first order Taylor expansion, including a bilateral filter inspired solution for cases where the surface depth difference is above a given threshold. These two approaches provide a normal estimation relative to the data source resolution and, additionally, the *online* method provides an efficient and robust estimation against sensor noise [13]. Notice that noisy and spiky surface data will affect the quality of the normal estimation step and, in turn, the downsampling step, decreasing the method efficiency and performance. In this regard, a normal estimation robust to noise is a basic part of the method, with a high impact on the matching results [27].

##### Downsampling

Traditional downsampling methods, also called subsampling or decimation, based on voxel-grid or Poisson-disk sampling, have a fixed size structure that do not consider local information and tend to either average or ignore parts of the data, removing and distorting important characteristics of the underlying surface. If these characteristics want to be somehow preserved, these methods require increasing the sampling rate, i.e., decrease voxel size, which in turn dramatically decreases the algorithm performance adding superfluous data. As an alternative to these problems, we propose a novel approach that accounts for the variability of the surface data without increasing non-discriminative pairs.

**Figure 6:**
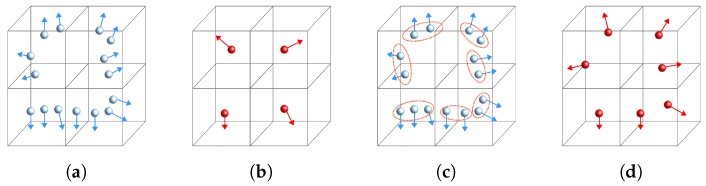
Representation of the normal clustering voxel-grid downsampling. (**a**) original cloud with a voxel-grid structure; (**b**) common downsampling by average; (**c**) novel proposed clustering approach; (**d**) result of the proposed clustering approach.

The proposed method is based on a novel voxel-grid downsampling approach using surface information and an additional non-discriminative pairs’ averaging step. The method starts by computing a voxel-grid structure for the point cloud data. For each voxel cell, a greedy clustering approach is used to group those points with similar normal information, i.e., the angle between normals is smaller than a threshold. Then, for each clustered group, we average the oriented points, effectively merging the similar points while keeping discriminative data. Figure 6 shows a simplified comparison between the common voxel-grid average method and the proposed normal clustering approach. Notice that, especially due to the PPF quantization space, for close points, distance is not relevant and normals encode the most discriminative information about underlying surface characteristics. As in the original method, the voxel size is set to $\Delta_{dist}$, defining a value relative to the model size. However, in our method, the parameter effect on the algorithm performance is significantly reduced, moving towards a more robust parameter-independent method.

Despite its local efficiency, this downsampling method does not account for the cases where the non-relevant surface characteristics are bigger than the voxel size. To mitigate these cases, when neighboring downsampled voxels contain similar data, we propose an additional step to average those points that do not provide additional surface information. This process is done by defining a new voxel-grid structure, with a much bigger voxel size (e.g., two or three times bigger), and averaging all points that do not have relevant normal data compared with all their neighbors’ voxels points. This step will reduce the points on planar surfaces, decreasing the number of total votes supporting the hypothesis. However, as the process is applied equally to the scene and the object, this will mainly decrease the votes of the non-discriminative parts, effectively increasing the value of the rest of the surface data.

#### 2.2.2. Feature Extraction

As mentioned before, Kiforenko et al. [27] published an exhaustive study and comparison of different types of PPF. Their results show that, despite the multimodal variants, the original four-dimensional feature [21] provides the best performance for range data. In light of this result, we propose to keep using the original PPF introduced in Section 2.1, represented in Figure 1 and Equation (1).

During the *Offline* stage, the model bounding box is obtained and the model diameter $d_{m}\in R$ is estimated as the diagonal length of the box. For a given PPF, a four-dimensional index is obtained using the quantization function defined in Equation (4):

(4) $$Q\left(x\right)={\left[\left\lfloor \frac{x_{1}}{\Delta_{dist}}\right\rfloor ,\left\lfloor \frac{x_{2}}{\Delta_{angle}}\right\rfloor ,\left\lfloor \frac{x_{3}}{\Delta_{angle}}\right\rfloor ,\left\lfloor \frac{x_{4}}{\Delta_{angle}}\right\rfloor \right]}^{T},$$

where the quantization step $\Delta_{dist}$ is set to $0.05\;d_{m}$ and $\Delta_{angle}$ is fixed to $\frac{\pi }{15}$. These values have been set as a trade-off between recognition rates and speed. In this way, the lookup table is defined with a size of $\left\lceil \frac{d_{m}}{\Delta_{dist}}\right\rceil \times \left\lceil \frac{\pi }{\Delta_{angle}}\right\rceil \times \left\lceil \frac{\pi }{\Delta_{angle}}\right\rceil \times \left\lceil \frac{\pi }{\Delta_{angle}}\right\rceil$. After preprocessing, for each model pair, the quantized PPF index is obtained and the reference point and the computed $\alpha_{m}$ angle are saved into the pointed table cell. In this case, all points of the model are used.

During the online stage, for each reference point, all possible point pairs will be computed and, using the four-dimensional lookup table, matched with the object model. Following the solution proposed by [21], only one of every five points (in input order) will be used as a reference point, while all points will be used as second points. To improve the efficiency of the matching part, in order to avoid considering pairs further away than the model diameter $d_{m}$, for each scene reference point, we propose to use an efficient Kd-tree structure to obtain only the second points within the model diameter.

#### 2.2.3. Matching

As explained in Section 2.1, the Point Pair Feature voting approach solves the matching problem by quantizing the feature space, grouping all similar pairs under the same four-dimensional index. As a result, any point pair is matched with all the other pairs that generate the same quantized features in a constant time. Despite its efficiency, this approach has two main drawbacks.

**Figure 7:**
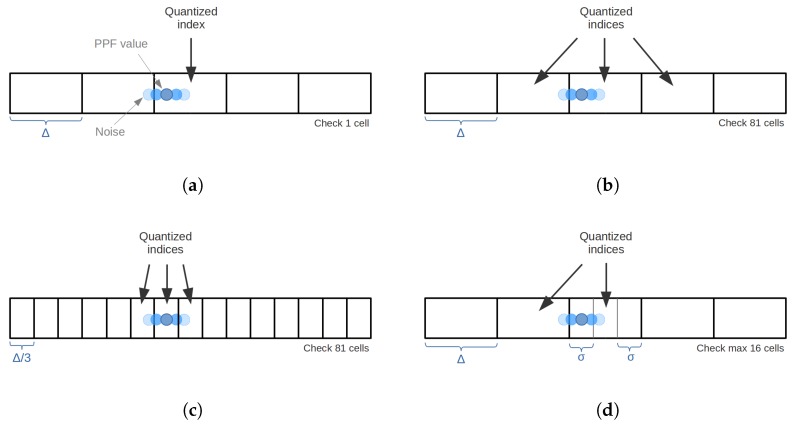
One-dimensional example of the noise effect on the lookup table during matching with four different strategies. (**a**) original approach; (**b**) approach of [26] using Δ; (**c**) approach of [26] using $\frac{\Delta }{3}$; (**d**) our approach.

The first drawback is regarding the noise effect on the quantized nature of the point pairs matching, as the quantization function *Q* can output different indices for very similar real values. In these cases, similar pairs generate different quantized index, which points to different cells of the lookup table, missing correct correspondences during the online stage. Figure 7a shows a one-dimensional representation of the problem. A straightforward solution was proposed by [26]. Their approach *spreads* the PPF quantized index to all its neighbors, effectively retrieving from the lookup table all the corresponding pairs pointed by the index alongside the pairs stored in its 80 neighboring cells, i.e., $3^{4}-1$ cells for a four-dimensional table. The main drawback with this method is the increased number of access to the lookup table, which is done for each matching PPF, decreasing significantly the time performance of the method. In addition, another problem can arise regarding the corresponding distance between features. If the quantization size Δ is kept, see Figure 7b, the correspondence distance increases, dramatically augmenting the number of corresponding pairs and introducing matching pairs with lower similarity level to the voting scheme. An alternative approach is to decrease the quantization size $\frac{\Delta }{3}$, see Figure 7c, accounting for the neighboring cells, using a bigger data structure.

We propose a more efficient solution by only checking a maximum of 16 neighbors keeping the size of the quantization step, as shown in Figure 7d. Considering that the difference between similar pairs are mainly generated by sensor noise, it is reasonable to assume that this noise follows a normal distribution characterized by a relatively small standard deviation σ, i.e., smaller than half of the quantization step $\sigma <\frac{\Delta }{2}$. Based on this assumption, we propose to check the quantization error $e_{q}=\left(\frac{x}{\Delta }-\left\lfloor \frac{x}{\Delta }\right\rfloor \right)\in \left[0,1\right)$ to determine which neighbors are more likely to be affected by the noise. This process is defined for each dimension by the piecewise function represented in Equation (5):

(5) $$N\left(e_{q}\right)=\left\{\begin{array}{cc} -1, & e_{q}<\frac{\sigma }{\Delta },\\ 1, & e_{q}>\left(1-\frac{\sigma }{\Delta }\right),\\ 0, & \mathrm{otherwise}, \end{array}\right.$$

where the result is interpreted as follows: -1 indicates that left neighbor could be affected, 1 indicates that right neighbor could be affected and 0 indicates that no neighbor is likely to be affected.

During matching, for each dimension, those pairs from neighbors that are likely to be affected by noise are retrieved. In practice, for generalization, we set the standard deviation value to three times the quantization step $\sigma =\frac{\Delta }{3}$; however, other values could be used regarding any specific noise model. This method have a best case scenario of accessing to a single table cell and worst case of accessing 16 cells, i.e., $2^{4}$. As we keep the same quantization step as the original method, a relatively lower similarity level correspondence may be retrieved during matching, yet with smaller number and negligible impact on performance.

The second drawback is related with multiple voting and over-representation of similar scene features. This problem is generated when during a scene reference point matching, several different pairs obtain the same combination of model correspondence and quantized α rotation angle. In detail, this happens when similar scene pairs obtain the same model correspondence and they have a similar scene angle value $\alpha_{s}$, generating the same quantized α index. Moreover, this situation is worsened by the neighboring checking method. This problem, especially found on planar surfaces, generates multiple superfluous votes for the same LC on the voting table that may produce a deviation in the results. Following the solution of [26], we avoid matching two model pairs with the same combination of quantized PPF index and scene angle $\alpha_{s}$. This process is efficiently done by creating an additional 32 bits variable for every PPF quantization index, where each bit represents a quantized value of the scene angle. In this way, when matching a point pair using a PPF, the bit value related to the scene angle is checked. Only if the bit is 0 is the matching allowed and the bit is set to 1, avoiding any new matching with the same exact combination. Notice that the first drawback could be more efficiently solved during training by duplicating the pairs on the neighboring cell. However, in this case, the second drawback will be more difficult to avoid, as keeping track of the same pairs on different cells will request a more complex checking strategy.

#### 2.2.4. Hypothesis Generation

As explained before, for each scene reference point, all the possible pairs are matched with the model. Then, during hypothesis generation, all consistent correspondence are grouped together generating a candidate pose. In detail, for each obtained scene–model pair correspondence, an LC combination is voted in the two-dimensional voting table. In this way, each position of the table represents an LC, which defines a model pose candidate in the scene, and its value represents the number of supports, which indicates how likely the pose is. The LC α angle is quantized by $\Delta_{angle}$ defining a voting table with a total size of $\left|M|\times \lceil \frac{2\pi }{\Delta_{angle}}\right\rceil$. After all votes have been cast, the highest value of the table indicates the most likely LC, defining a candidate pose for this scene reference point. At this step, an important problem arises from the assumption that a local coordinate always exists and, therefore, each piece of scene data has a corresponding model point. In reality, most scenes will have a majority of points that do not belong to the object. In order to avoid generating false positive poses, which can induce bias to the following clustering step, we propose defining a threshold to only consider LC with a minimum number of supports, e.g., three or five votes. Therefore, if the peak of the table is below this number, the pose will be discarded; otherwise, a candidate pose with an associated score is generated.

#### 2.2.5. Clustering

The matching result of different scene reference points yields multiple candidate poses which may be defining the same model hypothesis pose. In order to joint similar poses together, we propose using a hierarchical complete-linkage clustering method. This clustering approach enforces that all combinations of elements of each cluster follow the same conditions based on two main thresholds, distance and rotation. In practice, we sort the candidate poses by their vote support and create a cluster for each individual pose. Then, all clusters are checked in order and two clusters are joined together when for all combinations of their elements the conditions hold. In this way, the most likely clusters will be merged first, reducing the effect of mutual exclusive combinations. In detail, for two defined thresholds θ and ω, two clusters $C_{i},C_{j}\subset SE\left(3\right)$ will be joined if they satisfy the condition:

(6) $$max\left\{dist\left(P_{k},P_{l}\right)|P_{k}\in C_{i},P_{l}\in C_{j}\right\}<\theta \wedge max\left\{rot\left(P_{k},P_{l}\right)|P_{k}\in C_{i},P_{l}\in C_{j}\right\}<\omega ,$$

where the binary function dist:SE(3)×SE(3)→R represents the Euclidean distance and the binary function rot:SE(3)×SE(3)→[0,π] represents the rotation difference between two poses defined by the double arccosine of the inner product of unit quaternions [31]. Finally, for each cluster, all elements are merged and individual scores are summed up to define a new candidate pose.

#### 2.2.6. Postprocessing

At this point, the method provides a list of candidate poses sorted by score. The score of each pose is just an approximation obtained from the sum of each clustered pose number of matching pairs. Due to the nature of the hypothesis generation and clustering steps, joining poses obtained from each table peak, the clustered pose score may not properly represent how well the pose fits the object model to the scene. In this regard, we propose computing a more reliable value through an additional re-scoring processes. This new score will be computed by adding the total number of model points that fit the scene, where a fitting point is a model point closer to a scene point than a threshold. In particular, for a given pose P∈SE(3), the fittings score is computed as shown in Equation (7):

(7) $$S_{fitting}\left(P\right)=\sum_{m\in M}[min\{||Pm-s|||s\in S\}<th],$$

where [] represents the Iverson bracket and *th* represents the maximum distance threshold. Taking into account the preprocessing of the data, this threshold is set to half of the voxel size. Notice that this re-scoring procedure can be efficiently solved by a Kd-tree structure.

Even though this process provides a better fitting value approximation, there are two important issues that can still reduce the accuracy of the score. First, the deviation produced by model points that are self-occluded in the scene by the camera view, and, second, the possible aligning error of the object model respect to the scene. In order to mitigate these problems, we propose to use an efficient variant of the ICP algorithm alongside a perspective rendering of the object model for each hypothesis pose. For every clustered pose, the model object will be rendered using a virtual camera representing the scene acquisition system. At this point, the rendered data will be downsampled in the same way than the scene data. After that, an efficient point-to-plane ICP algorithm, based on Linear Least-Squares Optimization [32], using projective correspondence [33] will be applied. Despite the efficiency of this process, the large number of hypotheses obtained from the previous steps could significantly affect the whole method performance. A compromise solution is to apply this re-scoring and ICP steps only to the subset of the clustered poses with the higher scores, which represent the more likely fitting poses.

**Figure 8:**
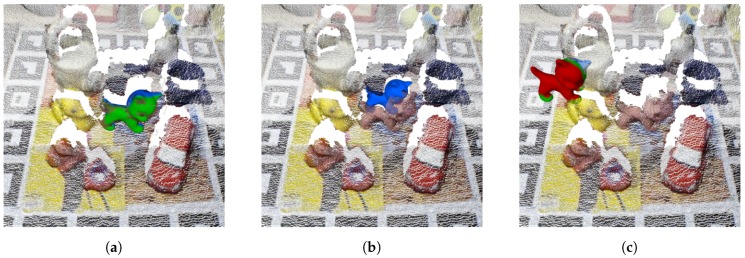
Model surface points classification regarding its distance to the scene data from the camera view. Green, blue and red colors label inlier, occluded and non-consistent points, respectively on the cat model surface. (**a**) matching pose; (**b**) occluded pose; (**c**) non-consistent pose.

Based on the ideas proposed by [23,25,26], after the re-scoring process, two verification steps are applied to filter false positive cases. These steps are introduced to discard well fitting model poses that do not consistently represent the underlying scene data. The first step checks the model-scene data consistency and discards cases which do not properly match the visibility context of the scene data. From the virtual camera point of view, each point of the rendered view of the model can be classified in three types, regarding its position with respect to the scene data: inlier, occluded and non-consistent. Inlier, shown in Figure 8a, is a model point that is near a scene point within a threshold distance and it is considered to match and explain the underlying scene surface. Occluded, shown in Figure 8b, is a point that is further away from the scene than a surface inlier; therefore, it is below the scene surface and can not be considered right or wrong. Non-consistent, shown in Figure 8c, is a point that is closer to the camera than a surface inlier, which means that it is not explained by the scene data and it is considered wrong. Hypotheses with a big percentage of occluded points or relatively small percentage of non-consistent points are likely to be false positive cases, hence discarded. In order to deal with challenging cases and certain degree of sensor noise, a maximum percentage of 15% of non-consistence points and 90% of occlusion is used.

**Figure 9:**
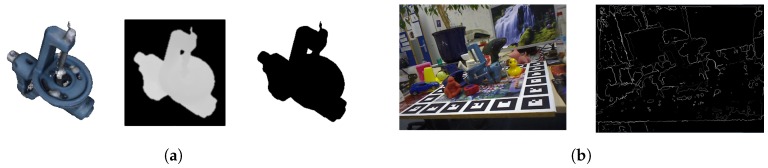
Examples of the extraction of the object model silhouette and the scene edges. (**a**) object model silhouette; (**b**) scene edges.

The second verification step accounts for well fitting poses with non-matching surface boundaries. This checking procedure is especially useful to discard cases relaying on planar or homogeneous surfaces without relevant surface characteristics, which can easily be incorrectly fitted to other similar scene surfaces if no boundary considerations are applied. For each hypothesis pose, this step extracts the silhouette of the object model from the camera view, as shown in Figure 9a, and compares it with scene extracted edges, Figure 9b. The scene edges are extracted by identifying depth and normal variations. The comparison is performed by averaging the distance from each silhouette point to the scene edges. Therefore, having a set of pixels defining the scene edges $E_{s}\subset Z^{2}$, for each different model pose view silhouette, defined by a set of pixels $E_{m}\subset Z^{2}$, the average edge score can be computed as:

(8) $$S_{edge}\left(E_{m},E_{s}\right)=\frac{1}{|E_{m}|}\sum_{e_{m}\in E_{m}}min\{||e_{m}-e_{s}\left|\right||e_{s}\in E_{s}\}.$$

Poses where the final score is higher than a threshold are discarded. In practice, a threshold of 5 pixels is used as an average distance error.

Notice that both steps may wrongly discard true positive cases under high occlusion. In this sense, both verification steps represent a trade-off between false positive pruning and occlusion acceptance rate. Hence, a request for high scene consistency, in terms of visibility context and contour matching, will reduce the capability of the system to handle occluded cases in benefit of higher reliability for normal cases.

## 3. Results

The method has been evaluated on the recently released BOP benchmark [29], first introduced in a workshop of the ICCV17 conference. The experimental results are divided into two distinctive parts: (1) method’s steps and parameter analysis and (2) performance evaluation against 15 state-of-the-art approaches.

### 3.1. Datasets

Hodan et al. [29] have recently proposed a complete evaluation benchmark for 6D pose estimation. Their work introduces an evaluation metric, an online platform and the combination of an extensive, variate and challenging sets of publicity available RGB-D datasets, tested with state-of-the-art methods. The benchmark, shown in Table 1, combines eight datasets including 89 object models and 62,155 test images with a total of 110,793 test targets. Each dataset is provided with textured-mapped 3D object models and training images from real or synthetic scenes. Notice that, for our method, only the 3D object model has been used without the texture information. The test images have distinct levels of complexity with occlusion and clutter including different types of objects, from common household objects to industrial-like pieces. For evaluation, the benchmark proposes to use a variation of the Visible Surface Discrepancy (VSD) evaluation metric [34], which is robust against ambiguous cases, explained in [29]. In this regard, all the presented results have been obtained using a misalignment tolerance τ=20 mm and correctness threshold θ=0.3. Due to the novelty of the benchmark, authors have published a subset of the original dataset to facilitate the comparison with the state-of-the-art and foster participation to the benchmark, in particular for slow methods. Based on the value of a robust evaluation metric and an extensive set of state-of-the-art results, we have tested our method on the aforementioned subset.

### 3.2. Steps and Parameter Analysis

Initially, the effect of different method’s steps and parameters are analyzed and compared. The main purpose of this section is to provide a picture of the method’s steps relevancy and the postprocessing parameter dependency of the proposed solution. As already mentioned, these tests has been conducted on the subset of datasets defined by the BOP benchmark [29]. If not explicitly indicated, all cases has been tested with the same parameters. The computational time difference between approaches is provided as a multiplication factor (e.g., two, three or four times slower) with respect to the faster approach in order to draw a more hardware-independent picture of the relation between recognition improvement and time cost.

**Figure 10:**
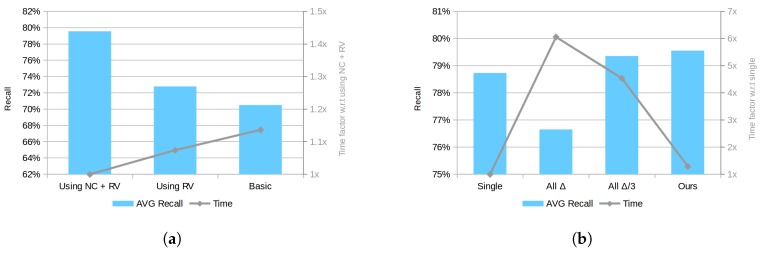
Performance comparison between different approaches. (**a**) comparison of the basic method against RV and NC improvements; (**b**) comparison between matching with single cell, all neighbors with Δ and $\frac{\Delta }{3}$ and our method using 16 cells maximum. Note: the left axis shows recall value in percentage and the right axis shows time factor.

First, the contribution of the proposed normal clustering downsampling step (NC), alongside the second averaging step, and the appropriateness of using a model rendered view (RV) for the re-scoring process are evaluated. In order to draw a clear picture of their contribution to the final method result, the two approaches have been disabled and their simpler approaches used. In detail, a common average voxel-grid and a whole model re-scoring process have been used as the basic alternatives. As can be observed in Figure 10a, using a rendered view (RV) for re-scoring, reduces the running time and provides a slightly higher recall, probably as a result of estimating a better fitting score using less data. In addition, when this part is combined with the proposed normal clustering (NC) approach for downsampling, the computational time further decreases and a very significant improvement in the results can be observed. Indeed, this result supports our previous reasoning that the preprocessing step is a key part of the method performance.

Second, the four discussed strategies for decreasing the effect of sensor noise on the quantization feature space are compared. Figure 10b shows the recall score for each approach and the time factor with respect to the single cell checking case. The results are surprising in several ways. On the one hand, it can be seen that the contribution of this part, analyzing the overall recall for the tested datasets, is relatively small with less than 1% improvement for all cases. On the other hand, in our implementation, the proposed solution performance goes beyond the designed efficiency and provides, on average, better results than the other approaches with dramatically lower time. Although this effect is irregular for different types of objects and scenes, a plausible explanation for these results can be attributed to the increased correspondence distance between PPF. Indeed, the proposed approach provides a larger distance only for limited cases, effectively only slightly increasing the overall distance, while avoiding the introduction of many matched pairs with a low similarity level to the voting scheme.

**Figure 11:**
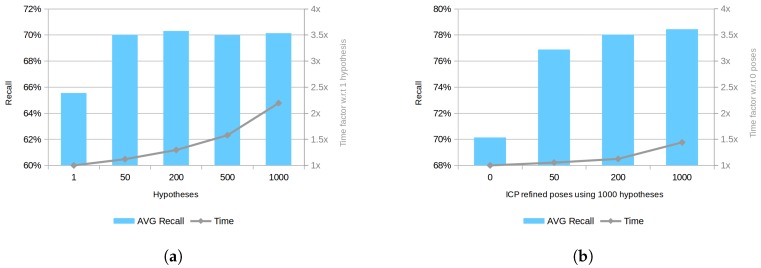
Performance comparison using different post-processing parameters. (**a**) comparison using a different number of hypotheses, no ICP or verification step is applied; (**b**) comparison refining different number of poses for 1000 hypotheses, any verification step is applied. Note: left axis shows recall value in percentage and right axis shows time factor.

Third, the effect of the method postprocessing parameters on the result has been studied by analyzing different cases. Initially, the effect of considering different number of hypotheses has been studied. Figure 11a shows the obtained average recall results for all datasets taking into consideration different number of hypotheses without using any ICP or verification step. As can be seen, the re-scoring process only accounts for a relatively small improvement with respect to the one hypothesis case, which represents the best hypothesis obtained after the clustering step. In addition, it can be observed that the re-scoring process alone does not provide any significant improvement using 50, 200, 500 or 1000 hypotheses. Following this direction, a test for analyzing the ICP effect into the re-scoring process using 1000 hypotheses has been conducted. Notice that the verification steps have not been used. As shown in Figure 11b, after poses are refined using ICP, the re-scoring process becomes more effective and performance increases with respect to the number of poses refined. This improvement is slowly decreasing for a higher number of poses, suggesting that, in fact, hypotheses are sorted by their likelihood, as expected. These results also corroborates the value of the ICP step to estimate a more accurate fitting score value.

### 3.3. Performance Evaluation

The proposed method has been evaluated against 15 state-of-the-art methods on the BOP Benchmark. These methods cover all main research branches with local feature-based, template matching and machine learning approaches. For a fair evaluation, all methods have been tested using the same fixed set of parameters for all objects and datasets. Notice that some of the methods also make use of RGB data (e.g., Drost-10-edge, Hodan-15, Branchmann-16 and Kehl-16). Additional details about the evaluation metric and tested methods can be found in [29].

**Figure 12:**
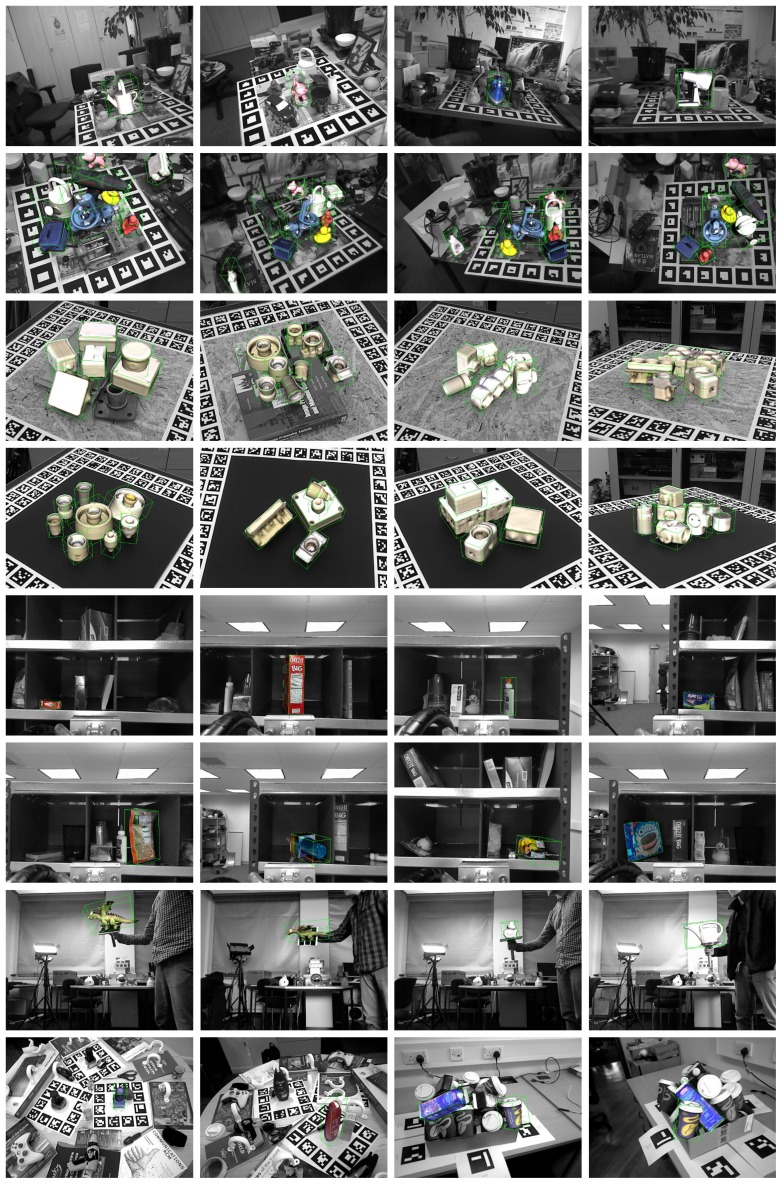
Proposed method results in scenes from the BOP benchmark datasets. Scene RGB data is shown in gray. Object models are shown in color and inside a green bounding box. Notice that, for scenes with multiple instances, only the most supported instance is used.

Based on the step and parameter evaluation part, the proposed method has been evaluated using 200 hypotheses refined by ICP. This setup, although not providing the best possible recall, represents a good trade-off between speed and recognition rates. Figure 12 shows some examples of the proposed method results. Table 2 shows the result of the comparison.

As can be seen from the obtained results, the proposed method outperforms the rest of the solutions, obtaining an average recall of 79.5%. In general, the proposed method performance surpasses the evaluated state-of-the-art with a relative improvement of more than 6% with respect to Vidal-18 [28]. For all datasets, the obtained average recalls show a significant improvement with respect to the state-of-the-art, with a very notable boost on *T-LESS* and *RU-APC*. In particular, for the *RU-APC* case, the proposed method obtains a relative improvement of 19%, moving from 37.83% obtained by Kehl-16 [20] to 44.92%. Overall, the obtained results show higher reliability for different types of objects, including household objects, e.g., *LM* or *TUD-L*, polygon shapes, e.g., *RU-APC*, and industrial-like pieces, e.g., *T-LESS*. In addition, results also suggest higher robustness against occluded scenarios, e.g., *LM-O* and *T-LESS*. Comparing different method types, the proposed method obtains the best recall within the feature-based approaches and outperforms the template matching and machine learning approaches. In detail, compared to the best feature-based approach, the preliminary work in [28], the more-discriminative preprocessing steps, improved re-scoring part and novel clustering approach shows a clear improvement for all datasets. Additionally, the proposed method moves Point Pair Features voting approaches away from the top template matching technique (Hodan-15 [14]), especially for *LM*, *IC-MI* and *RU-APC* datasets. Similarly, the method recall also improves with respect to the top machine learning technique (Brachmann-16 [18]), in particular for the *TUD-L* dataset, for which previously this method had the highest recall. Regarding time performance, the proposed method has an average execution time of 0.99 seconds per target on an Intel i7-5930K. Notice that this performance is obtained without using GPU. Finally, we would like to notice that conclusions regarding the different methods’ performance obtained from the benchmark [29] are significantly different than those of previous results presented in state-of-the-art. In detail, comparing the evaluations presented in [14,17,18], results lead to different conclusions, especially regarding the performance of the method proposed by Drost et al. [21], which seems underestimated in those previous cases. We attribute this discrepancy to the improved quality of the benchmark [29] with respect to the previous evaluation procedure, including a fixed training and testing framework with wider object domain and increased number of testing targets, fixed parameter requirements and improved evaluation metric. For these reasons, we did not evaluate the presented method against some other related approaches, like [26], which used this previous evaluation procedure.

## 4. Conclusions

In this paper, a method for 6D pose estimation of free-form rigid object based on the Point Pair Features voting approach [21] has been proposed for range data. The method introduces a novel preprocessing step, which considers the discriminative value of surface information, alongside an improved matching method and a hierarchical complete-linkage clustering approach. In addition, a novel view-dependent re-scoring process and two scene consistency verification steps are proposed to improve performance and decrease false-positive cases. The method’s steps and postprocessing parameters are analyzed, showing the improvement of the proposed steps and the efficiency of the method. Finally, the performance of the method is evaluated against 15 state-of-the-art solutions on a set of extensive and variate publicly available datasets. The presented results show that the proposed method outperforms all the other methods for all datasets, obtaining an overall average recall of 79.5%.

References

## Figures and Tables

**Table 1:** BOP benchmark dataset [29]. Each dataset has several objects, training images and test images. Some test images have more than one object, defining several test targets. *Used* values represents the current subsets used for the methods evaluation.

Dataset	Objects	Training Images		Test Images		Test Targets	
		**Real**	**Synt.**	**Used**	**All**	**Used**	**All**
LM	15	-	1313	3000	18,273	3000	18,273
LM-O	8	-	1313	200	1214	1445	8916
IC-MI	6	-	1313	300	2067	300	2067
IC-BIN	2	-	2377	150	177	200	238
T-LESS	30	1296	2562	2000	10,080	9819	49,805
RU-APC	14	-	2562	1380	5964	1380	5911
TUD-L	3	>11,000	1827	600	23,914	600	23,914
TYO-L	21	-	2562	-	1680	-	1669
Total	89			7450	62,155	16,951	110,793

**Table 2:** Recall scores (%) for the BOP Benchmark [29] using the VSD metric with τ=20 mm and θ=0.3.

Method	LM	LM-O	IC-MI	IC-BIN	T-LESS	RU-APC	TUD-L	AVG
Our method	**90.73**	**61.87**	**98.67**	**97.50**	**72.14**	**44.92**	**90.67**	**79.50**
Vidal-18 [28]	87.83	59.31	95.33	96.50	66.51	36.52	80.17	74.60
Drost-10-edge [35]	79.13	54.95	94.00	92.00	67.50	27.17	87.33	71.73
Drost-10 [21,35]	82.00	55.36	94.33	87.00	56.81	22.25	78.67	68.06
Hodan-15 [14]	87.10	51.42	95.33	90.50	63.18	37.61	45.50	67.23
Brachmann-16 [18]	75.33	52.04	73.33	56.50	17.84	24.35	88.67	55.44
Hodan-15-nr [18]	69.83	34.39	84.67	76.00	62.70	32.39	27.83	55.40
Buch-17-ppfh [36]	56.60	36.96	95.00	75.00	25.10	20.80	68.67	54.02
Kehl-16 [20]	58.20	33.91	65.00	66.00	35.95	37.83	38.67	47.94
Buch-17-si [36]	33.33	20.35	67.33	59.00	13.34	23.12	41.17	36.81
Brachmann-14 [17]	67.60	41.52	78.67	24.00	0.25	30.22	0.00	34.61
Buch-17-ecsad [36]	13.27	9.62	40.67	59.00	7.16	6.59	24.00	22.90
Buch-17-shot [36]	5.97	1.45	43.00	38.50	3.83	0.07	16.67	15.64
Tejani-14 [19]	12.10	4.50	36.33	10.00	0.13	1.52	0.00	9.23
Buch-16-ppfh [37]	8.13	2.28	20.00	2.50	7.81	8.99	0.67	7.20
Buch-16-ecsad [37]	3.70	0.97	3.67	4.00	1.24	2.90	0.17	2.38

## References

[1] Andreopoulos A., Tsotsos J.K. (2013). 50 Years of object recognition: Directions forward. Comput. Vis. Image Underst..

[2] Ullman S. (1990). Three-dimensional object recognition. Cold Spring Harb. Symp. Quant. Biol..

[3] Nevatia R., Binford T.O. (1977). Description and recognition of curved objects. Artif. Intell..

[4] Besl P.J., Jain R.C. (1985). Three-dimensional Object Recognition. ACM Comput. Surv..

[5] Sansoni G., Trebeschi M., Docchio F. (2009). State-of-The-Art and Applications of 3D Imaging Sensors in Industry, Cultural Heritage, Medicine, and Criminal Investigation. Sensors.

[6] Guo Y., Bennamoun M., Sohel F., Lu M., Wan J. (2014). 3D Object Recognition in Cluttered Scenes with Local Surface Features: A Survey. IEEE Trans. Pattern Anal. Mach. Intell..

[7] Horn B.K.P. (1984). Extended Gaussian images. Proc. IEEE.

[8] Rusu R.B., Bradski G., Thibaux R., Hsu J. Fast 3D recognition and pose using the Viewpoint Feature Histogram. Proceedings of the 2010 IEEE/RSJ International Conference on Intelligent Robots and Systems.

[9] Wohlkinger W., Vincze M. Ensemble of shape functions for 3D object classification. Proceedings of the 2011 IEEE International Conference on Robotics and Biomimetics.

[10] Aldoma A., Vincze M., Blodow N., Gossow D., Gedikli S., Rusu R.B., Bradski G. CAD-model recognition and 6DOF pose estimation using 3D cues. Proceedings of the 2011 IEEE International Conference on Computer Vision Workshops (ICCV Workshops).

[11] Steger C. (2002). Occlusion, clutter, and illumination invariant object recognition. Int. Arch. Photogramm. Remote Sens..

[12] Hinterstoisser S., Lepetit V., Ilic S., Fua P., Navab N. Dominant orientation templates for real-time detection of texture-less objects. Proceedings of the 2010 IEEE Computer Society Conference on Computer Vision and Pattern Recognition.

[13] Hinterstoisser S., Holzer S., Cagniart C., Ilic S., Konolige K., Navab N., Lepetit V. Multimodal templates for real-time detection of texture-less objects in heavily cluttered scenes. Proceedings of the 2011 International Conference on Computer Vision.

[14] Hodaň T., Zabulis X., Lourakis M., Obdržálek Š., Matas J. Detection and fine 3D pose estimation of texture-less objects in RGB-D images. Proceedings of the 2015 IEEE/RSJ International Conference on Intelligent Robots and Systems (IROS).

[15] Kotsiantis S.B. (2007). Supervised Machine Learning: A Review of Classification Techniques. Informatica.

[16] Bishop C.M. (2006). Pattern Recognition and Machine Learning.

[17] Brachmann E., Krull A., Michel F., Gumhold S., Shotton J., Rother C., Fleet D., Pajdla T., Schiele B., Tuytelaars T. (2014). Learning 6D Object Pose Estimation Using 3D Object Coordinates. Computer Vision—ECCV 2014.

[18] Brachmann E., Michel F., Krull A., Yang M.Y., Gumhold S., Rother C. Uncertainty-Driven 6D Pose Estimation of Objects and Scenes from a Single RGB Image. Proceedings of the 2016 IEEE Conference on Computer Vision and Pattern Recognition (CVPR).

[19] Tejani A., Tang D., Kouskouridas R., Kim T.K., Fleet D., Pajdla T., Schiele B., Tuytelaars T. (2014). Latent-Class Hough Forests for 3D Object Detection and Pose Estimation. Computer Vision—ECCV 2014.

[20] Kehl W., Milletari F., Tombari F., Ilic S., Navab N., Leibe B., Matas J., Sebe N., Welling M. (2016). Deep Learning of Local RGB-D Patches for 3DObject Detection and 6D Pose Estimation. Computer Vision—ECCV 2016.

[21] Drost B., Ulrich M., Navab N., Ilic S. Model globally, match locally: Efficient and robust 3D object recognition. Proceedings of the 2010 IEEE Computer Society Conference on Computer Vision and Pattern Recognition.

[22] Wahl E., Hillenbrand U., Hirzinger G. Surflet-pair-relation histograms: A statistical 3D-shape representation for rapid classification. Proceedings of the Fourth International Conference on 3-D Digital Imaging and Modeling.

[23] Kim E., Medioni G. 3D object recognition in range images using visibility context. Proceedings of the 2011 IEEE/RSJ International Conference on Intelligent Robots and Systems.

[24] Drost B., Ilic S. 3D Object Detection and Localization Using Multimodal Point Pair Features. Proceedings of the 2012 Second International Conference on 3D Imaging, Modeling, Processing, Visualization Transmission.

[25] Birdal T., Ilic S. Point Pair Features Based Object Detection and Pose Estimation Revisited. Proceedings of the 2015 International Conference on 3D Vision.

[26] Hinterstoisser S., Lepetit V., Rajkumar N., Konolige K., Leibe B., Matas J., Sebe N., Welling M. (2016). Going Further with Point Pair Features. Computer Vision—ECCV 2016.

[27] Kiforenko L., Drost B., Tombari F., Krüger N., Buch A.G. (2018). A performance evaluation of point pair features. Comput. Vis. Image Underst..

[28] Vidal J., Lin C., Martí R. 6D pose estimation using an improved method based on point pair features. Proceedings of the 2018 4th International Conference on Control, Automation and Robotics (ICCAR).

[29] Hodan T., Michel F., Brachmann E., Kehl W., Buch A., Kraft D., Drost B., Vidal J., Ihrke S., Zabulis X. (2018). BOP: Benchmark for 6D Object Pose Estimation. ECCV.

[30] Dai J.S. (2015). Euler–Rodrigues formula variations, quaternion conjugation and intrinsic connections. Mech. Mach. Theory.

[31] Huynh D.Q. (2009). Metrics for 3D Rotations: Comparison and Analysis. J. Math. Imag. Vis..

[32] Low K.L. (2004). Linear Least-Squares Optimization for Point-to-Plane icp Surface Registration.

[33] Rusinkiewicz S., Levoy M. Efficient variants of the ICP algorithm. Proceedings of the Third International Conference on 3-D Digital Imaging and Modeling.

[34] Hodaň T., Matas J., Obdržálek Š., Hua G., Jégou H. (2016). On Evaluation of 6D Object Pose Estimation. Computer Vision—ECCV 2016 Workshops.

[35] MVTec HALCON. https://www.mvtec.com/halcon/.

[36] Buch A.G., Kiforenko L., Kraft D. Rotational Subgroup Voting and Pose Clustering for Robust 3D Object Recognition. Proceedings of the 2017 IEEE International Conference on Computer Vision (ICCV).

[37] Buch A.G., Petersen H.G., Krüger N. (2016). Local shape feature fusion for improved matching, pose estimation and 3D object recognition. SpringerPlus.

